# Foreign language enjoyment in language learning from a positive psychology perspective: a scoping review

**DOI:** 10.3389/fpsyg.2025.1545114

**Published:** 2025-04-30

**Authors:** Wenjie Wu, Muhammad Kamarul Kabilan

**Affiliations:** ^1^School of Educational Studies, University Sains Malaysia, Penang, Malaysia; ^2^Faculty of Letters, Universitas Negeri Malang, Malang, Indonesia

**Keywords:** foreign language enjoyment, positive psychology, language learning, second language acquisition, scoping review

## Abstract

The language learning process is inherently rich in emotional experiences. With the affective turn toward positive emotions in second language acquisition (SLA), foreign language enjoyment (FLE) has gained increasing scholarly attention over the past decade due to its pivotal role in facilitating language learning and improving learners' emotional wellbeing. However, previous studies on FLE have largely adhered to ontological, epistemological, and methodological orthodoxy, often approaching FLE from a static, monolithic, and linear perspective. Moreover, much of this research was conducted without explicitly grounding FLE within the framework of positive psychology (PP). In response to these issues, this scoping review aims to provide an innovative examination of FLE through the lens of PP and to provide novel insights into its role in SLA. By evaluating 36 studies on FLE within the framework of PP, this article highlights the dynamic, interactive, and context-sensitive nature of emotions. It synthesizes key research themes and contextual and methodological characteristics while identifying current research gaps. Finally, this review presents implications and provides recommendations for future relevant research in this area.

## 1 Introduction

Emotions such as anxiety, boredom, and enjoyment, which are prevalent in language classrooms (Bown and White, 2010), have been found to affect learners' language learning and second language (L2) performance by directing learners' attention and cognition, evoking and sustaining their interest in classroom activities, activating different modes of processing information, and influencing their engagement and self-regulation in language learning (Pekrun, 2006). The past three decades have witnessed rapid developments in research exploring emotions in SLA (Saito et al., 2018). Despite a wide range of studies on emotions, early emotional research has primarily focused on the impact of negative emotions on language learning, concentrating exclusively on L2 anxiety (e.g., MacIntyre, 2016; Shao et al., 2019). Given that language learners experience a diversity of emotions during language learning, there is a crucial need to adopt a holistic perspective to explore both negative and positive emotions in the language classroom.

In response to the call for a holistic view of a rich variety of emotions in SLA (Imai, 2010), more and more L2 researchers have begun to shift from exclusive attention to learners' anxiety toward the integration of both positive and negative emotions (Jiang and Li, 2017; Dewaele and Li, 2018). This new research trend aligns with the flourishing Positive Psychology (PP) movement in SLA, which highlights the importance of strengthening positive emotions and mitigating negative emotions to achieve a balance between the two (Wang and Marecki, 2021). Therefore, influenced by the positive transition in the domain of psychology, an affective turn in the field of SLA has also occurred, shifting focus from a predominant concern with negative emotions to positive emotions (Prior, 2019). As such, in the context of the PP movement, an increasing number of studies have empirically explored the positive emotions experienced by language learners during the learning process (e.g., Alrabai, 2021; Shao et al., 2020).

Among a large array of positive emotions experienced by L2 learners in the language classroom, enjoyment is the most commonly researched positive emotion across different contexts (Dewaele and Li, 2020; Piniel and Albert, 2018). The concept of foreign language enjoyment (FLE) was introduced into SLA by Dewaele and MacIntyre (2014). FLE, as the most prevalent and crucial positive emotion, is conceptualized as “the pleasant feelings that originate from going beyond homeostatic boundaries as well as extending oneself to gain new experiences, particularly when one encounters challenging tasks” (Elahi Shirvan et al., 2020, p. 2). In a similar vein, Botes et al. (2021) conceptualized FLE as a specific positive emotion experienced by learners in the language learning process, especially when they break through their limitations to accomplish difficult learning tasks and their psychological needs are satisfied in classrooms. Boudreau et al. (2018) distinguish enjoyment from pleasure by pinpointing that FLE “takes on additional dimensions such as intellectual focus, heightened attention, and optimal challenge” (p. 153). Previous studies have explored the structure of FLE in different educational and cultural contexts (e.g., Dewaele and MacIntyre, 2019; Li et al., 2020) and investigated the potential predictors of FLE (e.g., Botes et al., 2024; Li et al., 2021). For example, FLE has been found to be influenced by factors such as learner personality (Botes et al., 2024), classroom environment (Li et al., 2021), and teacher variables (Dewaele et al., 2018). In addition to the studies related to the exploration of the FLE construct as well as its antecedents in diverse contexts (Li et al., 2018), there is a wide range of studies investigating the relationship between FLE and other individual difference variables in SLA, like language mindset (Wang et al., 2021), motivation (Alrabai, 2021), and classroom environment (Li et al., 2022).

Despite the insights yielded from prior literature, much of the research into FLE has been characterized by ontological and epistemological orthodoxy (Wang et al., 2024). Ontologically, previous FLE studies have separated emotions, personal traits, cognitions, and contexts. In other words, these studies have investigated emotional, psychological, individual, and contextual variables in isolation from one another. Epistemically, these studies have conceptualized FLE as a static, linear, and homogeneous phenomenon that overlooks the fleeting, dynamic, and idiosyncratic characteristics of emotions (Li et al., 2024). In terms of methodological approaches, most studies have tended to examine FLE without considering its temporal properties. In other words, these studies are characterized by low sampling rates to examine FLE, resulting in a limited understanding of the temporal breadth of FLE experiences over time (Wang et al., 2024).

Although these existing studies have produced rich and valuable findings, FLE is still far from being adequately investigated. Therefore, considering the pivotal role of FLE in facilitating L2 learners' acquisition of a target language (Dewaele and Alfawzan, 2018), it is crucial to note the scarcity of research on FLE from an alternative and innovative perspective. This article aims to assess the current work and clearly delineate the prior literature within the PP paradigm to provide a critical overview of FLE, connect present studies to future research, and offer directions for FLE research. Consequently, in light of the review's purposes, the present study aims to address the following three questions:

RQ1: What are the research themes of the existing FLE studies from PP?
RQ2: What are the contextual and methodological characteristics of the FLE studies from PP?
RQ3: What research gaps should be considered for future studies on FLE from PP?

## 2 Literature review

### 2.1 Foreign language enjoyment in SLA

Despite the loose attention to enjoyment in the realm of language learning decades ago (e.g., Green, 1993; Brantmeier, 2005), FLE was officially introduced into the field of SLA by Dewaele and MacIntyre (2014). Furthermore, they developed the Foreign Language Enjoyment Scale, which includes 21 items. Later, Dewaele and MacIntyre (2016, p. 216) reconceptualized FLE as a “complex emotion, capturing interacting dimensions of challenge and perceived ability that reflect the human drive for success in the face of difficult tasks.” In the meantime, they simplified the original scale by reducing it to 14 items, which were categorized into two dimensions (i.e., FLE-social and FLE-private). The former was defined as “positive feelings, encouraging peers, nice teachers, and a supportive environment,” while the latter was known as “thoughts and feelings coalescing around a sense of accomplishment” (Dewaele and MacIntyre, 2016, p. 228). Moreover, Dewaele and Dewaele (2017) adapted the original two-dimensional structure and added one more dimension (i.e., peer-controlled vs. teacher-controlled positive atmosphere) to the construct of FLE. The existing literature has extensively substantiated FLE as a multifaceted and complex construct (Dewaele and MacIntyre, 2016).

Ever since the seminal words of Dewaele and MacIntyre (2014, 2016), numerous studies have been conducted on Foreign Language Enjoyment (FLE) in Second Language Acquisition (SLA). These studies primarily investigate FLE from various perspectives. First, research on FLE has demonstrated that it is a dynamic construct, exhibiting constant variations due to both internal and external learner factors (e.g., Dewaele et al., 2018; Jiang and Dewaele, 2019; Dewaele and Dewaele, 2020). For instance, learners' personality traits (Bensalem et al., 2023), emotional intelligence (Li, 2020; Li et al., 2021), and self-perceived communicative competence (Jiang and Dewaele, 2019) have been identified as significant antecedents of FLE. Additionally, learners' sociobiographical variables, such as age, gender, and prior experience, have also been found to influence FLE (e.g., Jiang and Dewaele, 2019; Dewaele, 2022). Furthermore, learners' attitudes have been shown to be crucial predictors of FLE; learners with positive attitudes toward English tend to experience more enjoyment (Dewaele, 2022). Moreover, external factors related to the learner, such as teacher characteristics, significantly affect FLE. Specifically, teacher friendliness and supportiveness (Li, 2022), teacher enthusiasm (Dewaele and Li, 2021), and teacher teaching style and skills (Zawodniak and Kruk, 2019) have a substantial impact on learners' FLE. In addition, teachers' personality traits, such as openness, extroversion, and agreeableness, have been shown to have a significantly positive relationship with learners' levels of FLE, while teachers' conscientiousness and neuroticism have no significant effect on learners' FLE (Ahmadi-Azad et al., 2020). The classroom environment (Khajavy et al., 2018), topic (Elahi Shirvan and Talebzadeh, 2018), and task (Chen, 2023) have also been found to play a vital role in predicting learners' FLE.

Other inquiries into FLE are mainly concerned with its relation to other individual variables. FLE, as a positive activating achievement emotion (Pekrun, 2006), has been shown to have a positive relationship with, or a mediating effect on, other desirable learning outcomes (Guo and Qiu, 2022). For example, prior studies have revealed that FLE has a positive relationship with learners' self-perceived language proficiency and academic achievement (Jin and Zhang, 2018; Li et al., 2020). In addition, FLE has been reported to be positively related to learners' L2 willingness to communicate (WTC) (Dewaele and Dewaele, 2017), engagement (Guo, 2021), and L2 grit (Elahi Shirvan et al., 2021). Moreover, FLE also plays a mediating role in influencing learners' emotional intelligence (Li, 2020), grit (Liu and Wang, 2021), and motivation and language performance (Zhang et al., 2020).

More recently, a new wave of research has emerged in the literature on emotions and their dynamic variations across diverse timescales from a dynamic perspective (Dörnyei and Ryan, 2015). FLE has been examined as a complex dynamic system that involves constant variations over time due to the interaction and interconnection of a series of heterogeneous factors (e.g., Dewaele and MacIntyre, 2016; Elahi Shirvan et al., 2020). For example, Elahi Shirvan and Talebzadeh (2020) adopted a complex dynamic approach to explore FLE at both macro (developmental course of language learning) and micro (individual language acts) levels (Wang et al., 2024). This study revealed both the trajectories of emotional development and the in-depth momentary variations, as well as the predicting sources, such as teacher variables and topics. Similarly, an increasing number of studies have explored the factors affecting the moment-to-moment variations of FLE, including learner-internal factors like motivation and linguistic factors, along with teacher variables such as teacher supportiveness (e.g., Boudreau et al., 2018; Elahi Shirvan and Talebzadeh, 2018; Saito et al., 2018).

Taken together, previous studies have shed light on the complex and dynamic nature of FLE, providing valuable insights into its various sources as well as its effects on other individual variables in language learning. Nonetheless, despite the mounting scholarly attention investigating FLE in the domain of SLA, there remains a noticeable scarcity of literature that presents comprehensive and systematic descriptions of FLE from a positive psychology perspective. The research on FLE was not well theorized (Dewaele and Li, 2020; Guo and Qiu, 2022) due to the fact that most researchers tend to conduct their studies and arrive at conclusions based on the empirical data collected from respondents. Even with the limited studies on FLE informed by certain theories, this small proportion of studies mostly adopted either control-value theory or broaden-and-build theory (e.g., Boudreau et al., 2018; Shao et al., 2019), which cannot theoretically provide a holistic and deep understanding of FLE in SLA. Hence, to facilitate an in-depth understanding of FLE, it is imperative to conduct additional ongoing review research, aiming to clarify the current research themes, methodological characteristics, and gaps of FLE from a positive psychology approach, to illustrate pedagogical implications and to offer recommendations for future directions of FLE research.

### 2.2 Positive psychology in SLA

Positive psychology is the scientific study that explores what goes right in life (Peterson, 2006) and what facilitates personal growth and individual wellbeing. It aims to cultivate positive human qualities and build personal strengths rather than merely identifying, preventing, and repairing weaknesses or illnesses (Seligman, 2002). The positive renaissance in the field of psychology has also catalyzed a positive turn in the field of SLA. Inspired by PP, MacIntyre and Gregersen (2012) were the pioneering scholars to apply PP in applied linguistics and argued that the power of language learners' positive emotions should be harnessed to facilitate language learning. Lake (2013) was one of the first to explicitly adapt and adopt PP concepts in his study of L2 learners' positive self in Japan (MacIntyre and Mercer, 2014, p. 158). Later, the special issue on “PP in SLA” was published in 2014, marking the advent of the PP movement in SLA and laying the foundations of PP in L2 research.

During the periphery stage of PP in SLA from 2012 to 2015, PP had remained under-researched due to the dominant role of the cognitive perspective (Sharwood Smith, 2017) and faced criticisms, as reflected by Lazarus (2003), who criticized: “(1) the dominant use of cross-sectional methods, (2) a tendency to employ a dichotomy of negative and positive emotions, (3) inadequate attention to differences among individuals and groups, and (4) unreliable measurement of emotions” (MacIntyre and Mercer, 2014, p. 160). To address these issues in PP, MacIntyre and Mercer (2014) highlighted the need to examine emotions using refined methodologies, such as analyzing emotions over different timescales, from the short term to the long term, and in an interacting and holistic manner, wherein negative and positive emotions can simultaneously occur and dynamically fluctuate. Additionally, they emphasized the importance of context and social variables in SLA. They also advocated for a perspective that compares group and individual differences in emotional experiences and pointed to the complex dynamic systems that align well with PP-inspired research to examine the interaction and variations of multiple variables over time. Despite the efforts of L2 researchers supporting the application of PP, one distinguishing feature in the early stage of PP is that it remained underexplored and marginalized in SLA.

The flourishing of PP research in SLA began in 2016 due to two seminal books on PP in SLA edited by MacIntyre et al. (2016) and Gabryś-Barker and Gałajda (2016), marking the official advent of PP in mainstream applied linguistics. Since then, PP has made significant theoretical contributions and has been widely applied in SLA. For example, Oxford (2016) expanded the “PERMA” model to develop her own new “EMPHATICS” model and provided a general description of PP in applied linguistics. Similarly, a broad range of topics and concepts arising from the two influential books were incorporated into the Three Pillars proposed by Seligman and Csikszentmihalyi (2000), including (1) positive language experiences at the learner-internal level (e.g., positive emotions and flow), (2) positive character traits at the individual level (e.g., emotional intelligence and self-efficacy), and (3) positive institutions at the contextual level (e.g., a positive classroom atmosphere). MacIntyre and Mercer (2014) later clarified and contextualized these topics within SLA. Specifically, from the PP perspective, researchers in SLA focus on (1) learners' positive experiences, including emotions, (2) learners' positive character traits, such as emotional intelligence, and (3) positive institutions that allow learners to flourish at the contextual level. Among the Three Pillars, learners' emotions in language classrooms, part of the first pillar, have garnered the most attention worldwide.

In addition, MacIntyre et al. (2019) put forward an agenda for PP research in SLA. PP encourages a perspective by examining negative and positive emotions in a dynamic and interacting manner rather than simply isolating them. Furthermore, language learning in a sociocultural context requires a balance of emotions at both the individual and group levels. Regarding epistemology, the researchers acknowledged the “empirical and theoretical plurality,” indicating that subsequent studies under PP were encouraged to utilize quantitative, qualitative, and mixed-method research designs (p. 269). Therefore, it is imperative to consider the research agenda advanced by MacIntyre et al. (2019) in the second flowering stage of PP. Most studies have begun to examine emotions across different time scales, confirming the interacting and interconnected nature of emotions, considering the influence of contexts at diverse levels, and adopting multiple epistemological stances and methodological designs (Dewaele et al., 2019). Additionally, more studies have explored constructs within the PP framework to understand both intra- and inter-individual variations to optimize language learners' strengths and wellbeing while also improving language learning (e.g., Elahi Shirvan and Talebzadeh, 2018; Gregersen and MacIntyre, 2017). Considering the second period of PP development, PP provides a balanced model for examining the interplay and interaction between positive and negative emotions in SLA and is well-suited to explore the complex, dynamic, and context-sensitive emotional episodes of language learners (Wang and Marecki, 2021).

Taken together, in contrast to the limitations of past research that examined emotions from a static, monothetic, and isolated view, positive psychology (PP) provides an alternative, innovative, and holistic perspective by exploring how enjoyment, a key construct from the first pillar, is integrated and interconnected with other topics, such as emotional intelligence (EI) from the second pillar and classroom climate from the third pillar. This approach leads to a more comprehensive understanding of learners' emotional experiences in second language acquisition (SLA). This study will provide a review of foreign language enjoyment (FLE) from the positive psychology perspective and offer fresh insights into FLE in SLA. The specific aspects of positive psychology examined in the reviewed studies mainly focus on its factors and tenets. More specifically, this review examines enjoyment as the most extensively researched positive emotion. Additionally, this study emphasizes the tenets explaining how positive emotional experiences promote language learners' learning and academic achievement through the interplay of the constructs under the Three Pillars, as well as how positive psychology is empirically applied to the area of SLA to facilitate second language (L2) learners' academic performance and enhance emotional wellbeing. Hopefully, this review can provide some pedagogical implications and shed insightful light on future applications of PP and emotional research.

## 3 Materials and methods

This study adopts a scoping review approach, which is a kind of literature review particularly suitable when research in a specific area has not yet been fully reviewed or when it involves complex and heterogeneous characteristics regarding research focus and methodological designs (Pham et al., 2014). Although scoping reviews share similarities with systematic reviews in procedural features, they are distinct in terms of their purposes and aims. More specifically, scoping reviews examine what a domain has accomplished and how relevant research is conducted in a certain field, thus providing an overview of the types of existing literature in that field of research (Arksey and O'Malley, 2005). Therefore, scoping reviews are generally used to assess patterns of knowledge and research designs from a broader range of studies (Levac et al., 2010).

The present scoping review seeks to achieve several objectives. First, as enjoyment has been confirmed as the most prevalent and salient positive emotion in language learning (Pavelescu and Petrić, 2018), this study aims to examine the research themes of FLE over the past decade. Second, it assesses the research contextual settings, such as participant characteristics and instructional environments in the existing studies. In addition, in light of the growing methodological guidance available, the present review intends to explore the methodological designs and contextual characteristics of previous empirical FLE research in SLA to identify features in research designs and approaches. Moreover, this study is also intended to identify the gaps and offer recommendations for future research and the development of FLE within PP. Given the insightful and invaluable findings from past studies, this review also attempts to highlight the significant contributions this body of research has made to the field, draw conclusions regarding the limitations of current research, and provide useful implications and directions for future research.

The present review searched for studies spanning the 10-year period of interest (2014–2024). This time range was chosen because the concept of FLE was first introduced into SLA in 2014 (Dewaele and MacIntyre, 2014). The search was conducted using the databases Web of Science (WOS) and SCOPUS to identify potentially relevant studies. These two databases were selected for this study because they are fundamental for social sciences (Gusenbauer and Haddaway, 2020), providing comprehensive coverage of pertinent peer-reviewed publications (Hallinger, 2020), and they help reduce bias while increasing the reliability and certainty of the reviewed studies (Page et al., 2021). Google Scholar (GS) was not included due to its lack of transparency, stability, precision, and control (Pranckute, 2021, p. 4). The researchers employed the search terms shown in Table 1. The search parameters involved the documented type “article” in the English language, and the entire text obtained was qualified. Review articles, conference papers, book chapters, and meta-analyses were excluded, as were articles not published in English. The screening criteria for this scoping review are shown in Table 2.

**Table 1:** Database search terms.

**Database**	**Search terms**
Web of Science (WOS)	(“foreign language enjoyment” OR “FLE”) AND (“Positive Psychology” OR “PP”) AND (“language learning” OR “second language learning” OR “SLA” OR “EFL” OR “English as a foreign language”)
SCOPUS	(“foreign language enjoyment” OR “FLE”) AND (“Positive Psychology” OR “PP”) AND (“language learning” OR “second language learning” OR “SLA” OR “EFL” OR “English as a foreign language”)

**Table 2:** Inclusion and exclusion criteria.

**Inclusion criteria**	**Exclusion criteria**
1. A full-text research article conducted in the field of language learning	1. Articles that are conducted in other languages or in other disciplines
2. Articles that involve an empirical design	2. Articles that are not accessible
3. Articles published in English	3. Methodological and conceptual articles were excluded
4. Articles explicitly adopting PP either individually or collectively with other theoretical frameworks	

**Figure 1:**
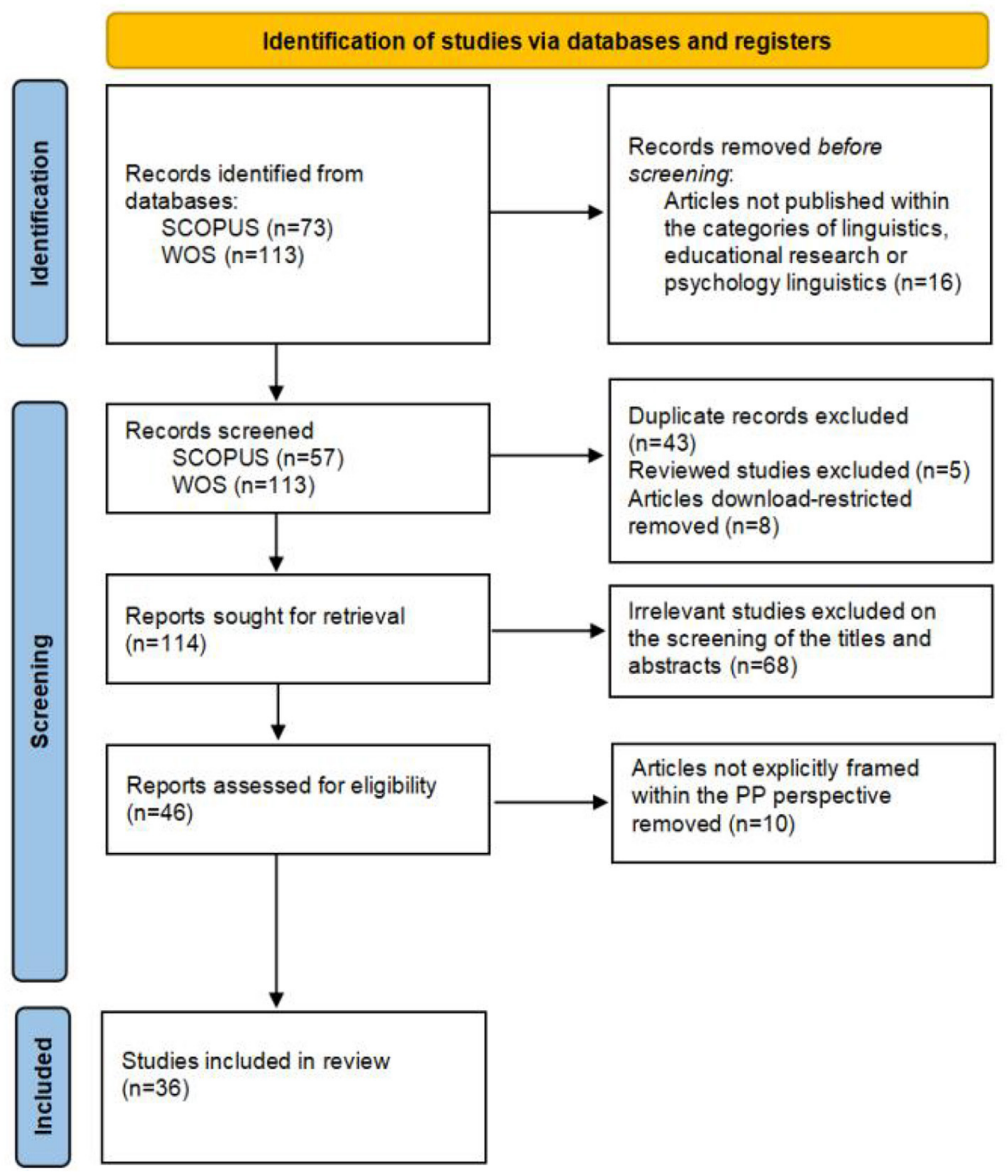
Flow diagram for scoping review (adapted from Page et al., 2021).

The entire searching and screening process is shown in Figure 1, which was in alignment with the Preferred Reporting Items for Systematic Review and Meta-Analysis (PRISMA) 2020 checklist (Page et al., 2021). According to the initial search, there were 113 articles from WOS and 73 articles from SCOPUS. Articles that were not published within the categories of linguistics, educational research, and psychological linguistics were excluded (*n* = 6). According to the screening process, 43 duplicates, eight download-restricted papers, and five reviewed articles were removed, which left 114 articles in total. Then, the remaining articles were separately reviewed by two researchers to determine the relevance of the research topic by looking at the titles, abstracts, and even the entire paper when necessary (Xiao and Watson, 2019). After the examination, 46 articles were left for further screening. Next, based on the screening criteria, the researchers chose articles that explicitly adopted PP to look at FLE and removed the papers that were not researched within PP. As a result, only 36 articles were qualified.

Content analysis was employed to assess the written content for this review study (Creswell, 2014). This process, which aimed to identify and determine the underlying meaning of the texts from the reviewed studies, took place at the interpretive level (Berg and Lune, 2017). The texts related to the research questions were coded by two researchers independently. The two researchers considered and determined the research themes of the reviewed studies. For the second research question, the researchers classified and summarized the contextual characteristics (i.e., country, target language, instructional setting, and participants' characteristics) and methodological characteristics (i.e., research design, sample size, data collection methods, research approach, and study length). This bottom-up coding technique enables the reviewers to provide accurate data from the studies without bias and subjectivity (Zou et al., 2022). The final coding results of the two researchers were compared by calculating the inter-rater reliability (Cohen's Kappa statistic, *k* = 0.80), which showed an almost perfect agreement level.

## 4 Results

This section synthesizes and analyzes the studies reviewed, published between 2014 and 2024, in response to the three research questions. To begin with, the research themes on FLE from PP were systematically categorized into distinct categories, as shown in Table 3. Additionally, based on the findings, the results for each theme were presented. Subsequently, the contextual and methodological characteristics of the evaluated studies were identified. Finally, the gaps that need consideration for future research on FLE from a PP perspective were also discussed.

**Table 3:** Research themes in the reviewed studies.

**Research themes**	**Examples**
The relationship with other variables	Willingness to communicate, emotional intelligence, engagement, motivation, learning achievement, classroom environment
The predictors of FLE	Learner variables, teacher variables, and contextual variables
The effect of FLE	Enhance willingness to communicate facilitate FL achievement promote learners' motivation increase learning engagement mitigate the negative effect of negative emotions
The nature of FLE	Dynamics of FLE measures of enjoyment dimensions of enjoyment
The emotional regulation of FLE	Emotion intelligence (EI) training cooperative learning

Some studies may include more than one aspect of FLE above.

### 4.1 RQ1: research themes

According to the reviewed studies, L2 researchers in SLA primarily conducted their FLE-related studies from five aspects, as shown in Table 3. These aspects include the relationship with other variables, the predictors of FLE, the effect of FLE, the nature of FLE, and the effectiveness of emotional intervention on FLE.

#### 4.1.1 The relationship with other variables

This category of research themes on FLE focuses on the relationship between FLE and other individual variables or PP components. Fifteen FLE studies (*n* = 15) (e.g., Lee et al., 2024; Dewaele and Pavelescu, 2021; Hosseini et al., 2022; Li and Xu, 2019) explored the relationships of FLE with other individual or contextual variables, such as willingness to communicate, engagement, and classroom environment. For example, Li (2020) examined the complex relationship between enjoyment, trait emotional intelligence (EI), and EFL learning achievement among Chinese high school students, revealing the indirectly mediating role of FLE between EI and learning achievement. Additionally, Hosseini et al. (2022) explored the relationship between enjoyment, engagement, and classroom climate, reporting a significantly positive relationship between FLE and engagement. Furthermore, Fathi and Hejazi (2024) and Wang et al. (2023) identified positive relations between FLE and learner achievement. Moreover, Dewaele and MacIntyre (2016) identified the relations between enjoyment and anxiety, positing that enjoyment and anxiety are not necessarily opposing emotions that function in a see-saw manner but can occur concurrently in the language learning process, which is in line with the findings from Dewaele et al. (2016).

#### 4.1.2 The predictors of FLE

A total of 13 studies (*n* = 13) explored the predictors or sources of FLE, including learner variables, teacher variables, and contextual variables (e.g., Dewaele et al., 2018; Jiang and Dewaele, 2019; Li, 2022). For instance, Dewaele et al. (2018) identified several learner-internal variables that are significant predictors of FLE, such as attitudes toward foreign language (FL) and language teachers, age, gender, and FL level, along with teacher-related variables like teacher predictability and FL use frequency. Moreover, Jiang and Dewaele (2019) also investigated the antecedents of FLE, including learner variables (i.e., self-perception of FL proficiency and attitudes toward English) and teacher variables (i.e., teacher strictness, teacher friendliness, and teacher humor). In addition, a range of contextual variables, such as tasks (Li and Dewaele, 2024) and classroom environment (Li et al., 2021), have also been reported as crucial antecedents of FLE.

#### 4.1.3 The effect of FLE

A total of 10 studies (*n* = 10) examined the effects of FLE during foreign language learning and language acquisition (e.g., Bensalem, 2022; Fattahi et al., 2023). More specifically, FLE has been shown to enhance learners' willingness to communicate in L2 (Bensalem, 2022), facilitate language learning achievement (Jin and Zhang, 2018), promote learners' motivation (Dewaele et al., 2023), increase L2 learners' engagement in learning (Derakhshan and Fathi, 2024), and mitigate the negative impacts of negative emotions such as boredom and anxiety (Dewaele and MacIntyre, 2016; Kruk et al., 2022; Li, 2022).

#### 4.1.4 The nature of FLE

Five studies (*n* = 5) explored the nature of FLE, including its dynamics (Chen, 2023; Elahi Shirvan et al., 2020), measures (Botes et al., 2021; Li et al., 2018), and dimensions (Jin and Zhang, 2018). Specifically, both Chen (2023) and Elahi Shirvan et al. (2020) identified the constantly dynamic characteristics of FLE across diverse timescales, both intrapersonally and interpersonally. Additionally, Li et al. (2018) examined the construct of FLE in a Chinese EFL context and confirmed the three-factor model of FLE, which includes FLE-private, FLE-teacher, and FLE atmosphere. Similarly, Jin and Zhang (2018) revealed three dimensions of FLE in an EFL context, encompassing English learning, teacher support, and student support. Moreover, a few studies have developed and validated instruments to measure enjoyment in FL classes. For example, inspired by Dewaele and MacIntyre's (2014) 21-item FLE scale, Botes et al. (2021) developed and validated the Short Form of the Foreign Language Enjoyment Scale (S-FLES), which includes only nine items.

#### 4.1.5 The emotion regulation on FLE

Only a small number of studies (*n* = 2) explored emotional regulation strategies to facilitate learners' FLE (Li and Xu, 2019; Zheng and Zhou, 2022). For example, Zheng and Zhou (2022) examined how learners' FLE was promoted and affected through cooperative learning, specifically positive goal interdependence and peer personal support in a classroom context. Similarly, Li and Xu (2019) also conducted emotional intervention research aimed at promoting learners' FLE through the “ARGUER” model of EI training.

### 4.2 RQ2: contextual and methodological characteristics

To address RQ2, this review study evaluated the contextual features across five aspects, including the distribution of countries, target language, learning setting, participant characteristics, and sample size. Additionally, the researchers reviewed the methodological characteristics across three aspects, including research design, research approach, and data collection instruments.

#### 4.2.1 Contextual characteristics

Table 4 presents an overview of the contextual characteristics identified in the reviewed studies. Regarding the distributions of countries, four countries were involved in the reviewed studies examining FLE from a PP lens in the field of SLA. A large number of the assessed studies on FLE in language learning were conducted in China (*n* = 24), with a few studies conducted in Iran (*n* = 8). Very few studies were undertaken in Turkey (*n* = 3) and Saudi Arabia (*n* = 2). In terms of the target language, all 36 studies (*n* = 36) aimed to explore the FL enjoyment of learners whose target language was English.

**Table 4:** Contextual characteristics.

**Contextual characteristics**	**Number**
**Distributions of countries**	
China	24
Iran	8
Turkey	3
Saudi Arabia	2
**Target language**	
English	36
**Learning setting**	
Generic instructed setting	33
Language for specific purposes	2
Blended setting	1
**Participant characteristics**	
University students	22
Senior high school	8
Junior high school	2
Primary school	1
Mixed-age groups	3
**Sample size**	
1–10	3
11–100	4
100–1,000	22
More than 1,000	7

As for the learning setting, according to the reviewed studies, it can be observed (Table 4) that a large number of studies were conducted in generic instructed settings (*n* = 33), with very few studies undertaken in the context of language for specific purposes (*n* = 2) and only one study in the blended setting (*n* = 1). Regarding participant characteristics, most studies (*n* = 22) chose university students as their samples, followed by senior high school students (*n* = 8), while junior high school students (*n* = 2) and primary school students (*n* = 1) were under-researched. There were a few studies (*n* = 3) selecting mixed-age groups as the sample, with participants ranging from high school students to adults. Concerning sample size, it was revealed that the sample sizes of most studies ranged from 100 to 1,000 participants (*n* = 22), followed by a few studies with sample sizes exceeding 1,000 (*n* = 7). There were four studies (*n* = 4) with sample sizes ranging from 11 to 100 and three studies (*n* = 3) with fewer than 10 samples.

#### 4.2.2 Methodological characteristics

As shown in Table 5, the methodological characteristics were summarized from three aspects: research approach, research designs, and data collection instruments. First, regarding the research approach, the quantitative research approach (*n* = 27) was the most commonly used method, as displayed in Table 5, followed by the mixed methods approach (*n* = 9). One noticeable feature is that no study employed a qualitative approach.

**Table 5:** Methodological characteristics.

**Methodological characteristics**	**Number**
**Research approach**	
Quantitative research design	27
Qualitative research design	/
Mixed-method design	9
**Research designs**	
Longitudinal approach	5
Cross-sectional approach	26
Not specified	5
**Data collection instruments**	
Questionnaire	35
Open-ended survey	8
Test	3
Interview	6
Reflective Journal	2

Focusing on studies that adopted quantitative and mixed-method approaches, the researchers further reviewed the research design. As shown in Table 5, the majority of the reviewed studies employed a cross-sectional design (*n* = 26), enabling L2 researchers to measure learners' FLE at a specific time point. In contrast, only a small number of studies (*n* = 5) utilized the longitudinal approach to examine the developmental trajectories of FLE over time. For example, Elahi Shirvan et al. (2020) explored the dynamism of different facets of FLE through a longitudinal design across various timescales, including seconds, minutes, weeks, and months in the EFL context. Five studies did not specify their research approach.

In terms of data collection instruments, the questionnaire was the most commonly used data collection method in the reviewed studies (*n* = 35), followed by the open-ended qualitative survey (*n* = 8). A few studies (*n* = 6) included interviews as a data collection method to probe into language learners' enjoyable experiences of learning. Three studies (*n* = 3) used tests to collect data, and only two studies (*n* = 2) utilized reflective journals or diaries as data collection methods to document learners' thoughts and experiences by asking them to reflect on their language learning.

### 4.3 RQ3: research gaps in FLE

Several studies have outlined the specific areas that are worth further investigation concerning the research on FLE in SLA. These research gaps have been grouped into three distinct categories.

#### 4.3.1 Expand the research scope of FLE

Among the 36 studies analyzed, the majority suggest expanding the research scope in future directions for FLE studies. These research areas can be classified into several aspects. First, L2 researchers (e.g., Wu and Halim, 2024; Li et al., 2023b) propose that more research is needed to conceptualize FLE across diverse language skill contexts, such as speaking and writing. Additionally, researchers highlight that very few empirical studies have examined how language teachers and practitioners can utilize the antecedents of FLE and certain strategies to help regulate and intervene in learners' FLE development to promote a positive learning experience (e.g., Elahi Shirvan et al., 2020; Bayat et al., 2020). Furthermore, few researchers have conducted studies to examine the relationship between FLE and other negative emotional constructs, as well as FLE's role in alleviating negative emotions within the PP framework (Wu, 2024). Moreover, it is suggested that future FLE research should focus on the transmission of FLE within the L2 classroom (Frenzel et al., 2018). Despite the limited research on emotional contagion in SLA (e.g., Talebzadeh et al., 2020), the exploration of the reciprocal nature of FLE transmission within teacher-student interactions in language classrooms remains inadequately addressed. Finally, there is a lack of research on FLE in various learning contexts, such as online learning contexts (e.g., Zheng and Zhou, 2022; Wang and Jiang, 2022) and AI-based learning classes (Zhang et al., 2024).

#### 4.3.2 Theorize the FLE in empirical studies

Despite the rich findings of FLE research from a PP lens in SLA, it should be noted that while the current review study examines FLE within the framework of PP, there is a new wave of research on FLE that integrates various theories, such as control-value theory, broaden-and-build theory, and complex dynamic systems theory, to gain a comprehensive and deep understanding of its nature, antecedents, and effects on language learning (Dewaele and Li, 2020). However, only a very limited number of studies have adopted an integrated theoretical perspective (Wang et al., 2021). Given the complex and dynamic nature of FLE, examining it solely from a single theoretical approach may not provide a holistic view of FLE, including how it interacts with other achievement emotions or contextual variables and how it changes over various timescales. The call for theoretical plurality in FLE research remains to be addressed (MacIntyre et al., 2019).

#### 4.3.3 Diversify the research designs and approaches

In terms of research designs and methods, existing studies on FLE mostly adopt quantitative research designs using dominant close-ended questionnaires to explore learners' emotions at a single time point. Very few studies employ mixed-method methodologies, and no pure qualitative empirical studies on this topic have been undertaken. Regarding emotion research in SLA, integrating quantitative and qualitative methods enables researchers to have binocular vision and allows them “to perceive three-dimensional images of phenomena” (Dewaele, 2019, p. 85). Therefore, it is advised that future studies on FLE consider using mixed methods by combining the rigorous statistics of quantitative methods with the in-depth insights of qualitative methods (e.g., Elahi Shirvan and Talebzadeh, 2018). Additionally, it is suggested that future studies on FLE expand research designs by adopting longitudinal approaches to collect data at multiple timescales, especially when researching the dynamic characteristics of emotions, such as the idiodynamic approach, retrodictive qualitative modeling, and Q-methodology, thus achieving richer methodological diversity (MacIntyre, 2016). Moreover, some researchers also pinpoint that to guarantee rigor and minimize bias in the research process, various types of triangulation, such as methodological triangulation, theory triangulation, and data triangulation, should be considered in future emotion research (Dewaele and Li, 2020).

## 5 Discussions

In this scoping review, the researchers first examined the themes of FLE studies informed by positive psychology from five perspectives and then summarized the contextual and methodological characteristics of this body of research. Moreover, this study also identified three research gaps that need to be addressed in the future.

### 5.1 Research themes

In terms of research themes, with the emergence of attempts to explore FLE from a PP lens, this review study finds that this body of research primarily focuses on the relationship between FLE and various individual and social variables, such as L2 WTC and engagement, as well as its predictive factors or sources of FLE, including learner-internal and -external sources. According to these studies, FLE is described as a complex and dynamic system influenced by a series of variables, which enhances learners' language learning and achievement (Jiang and Dewaele, 2019). Although these studies provide insights into modeling the relationship between FLE and other variables and exploring potential influential predictors of FLE, they generally suffer from a prevailing individual-oriented assumption, conceptualizing enjoyment as an intrapsychic construct in response to individual and contextual stimuli within the complex language learning process (Imai, 2010). In other words, these studies overlook the intrapersonal and interactive nature of emotions, which has not adequately applied the principles of PP to FLE research. Given the interactive nature of emotions and the socio-culturally constructed classrooms, it is imperative to examine how enjoyment develops through the discourse of teacher-student or student-student interaction within the language learning context.

Another concern regarding the themes of the relationship between FLE and other emotions requires further research attention. Although a limited number of studies have provided only a basic understanding of how enjoyment interacts with other types of emotions, primarily focusing on anxiety, the language classroom is filled with a wide range of emotions (Imai, 2010; Sampson, 2022), even within the same classes or tasks. Therefore, more scholarly attention should be directed toward the connections between enjoyment and a diverse array of emotions, investigating how these emotions reciprocally affect each other and develop into specific emotional phenomena during language learning in the future.

Additionally, positive psychology aims to promote wellbeing by enhancing the five key areas of “PERMA” (i.e., Positive Emotions, Engagement, Relationships, Meaning, and Achievement) (Seligman, 2011). However, current FLE research has not examined how FLE interacts with other elements of the PERMA model and collectively influences L2 learners' wellbeing. Furthermore, these FLE studies have not fully explored learners' positive experiences and character traits according to the Three Pillars.

In addition, only a small handful of studies have focused on the effect of FLE on the academic achievement of foreign languages. Even with the few reviewed studies that focus on the effect of FLE on language academic achievement, most of them were merely associated with its effect on writing achievement or general language performance (e.g., Li et al., 2023b; Wu and Halim, 2024). There is no research on the effect of FLE on other specific language skills, such as listening, speaking, or reading achievement. As Li et al. (2023a) pointed out, emotions have the characteristic of L2 skill specificity, which aligns with the findings from Dewaele and Li (2020) that enjoyment has different levels of effect on different language skills. Hence, the emotional experiences within different language skill contexts may differ due to the distinctive differences in language goals, cognitive necessity, language recursiveness and evanescence, and self-paced learning of distinct language skills (Li et al., 2024). Therefore, there is a crucial need to undertake more research on the role of FLE in different language skill contexts and examine their differences regarding the effect of FLE on various language skills.

Moreover, according to the results, only one reviewed study focused on examining the FLE in a distinctive blended learning context (Chen and Kim, 2023), which revealed that language learners experienced a relatively higher level of FLE than foreign language anxiety in a blended English learning context. Despite the valuable findings, current FLE-related research in diverse learning settings is very scarce and requires more empirical studies in the future. In different learning contexts, language learners may experience various emotions due to environmental or contextual influences. Additionally, the reciprocal interaction and connection between individual variables and specific contextual variables may contribute to differing patterns of emotional experiences (Li et al., 2023a). Building on the positive institutions at the group level of the Three Pillars to facilitate learner flourishing and promote wellbeing, these studies have not explored the impact of the facilitative sociocultural environment at the contextual level on learners' FLE. Therefore, more studies are needed to investigate FLE within PP in diverse instructional settings, such as online cooperative language learning and AI-assisted language learning, or with specific teaching models like Task-Based Language Teaching (TBLT) and Project-Based Language Teaching (PBLT).

Moreover, previous studies rarely focused on investigating the use of emotion regulation strategies to enhance FLE. Emotion regulation, as a key topic in positive psychology, can be employed to reduce negative emotions that hinder language performance or to facilitate positive emotions that enhance learners' efficacy (Greenier et al., 2021). However, very few studies have explored the effectiveness and mechanisms of emotion regulation strategies in reducing negative emotions, facilitating FLE, and enhancing learners' engagement and achievement from the PP perspective (Mercer et al., 2018). Given that emotion regulation significantly impacts personal psychological wellbeing and academic achievement (Li et al., 2024), more research is needed on enhancing enjoyment and alleviating negative emotions through effective emotion intervention strategies in SLA. This aligns with Oxford's (2016) “EMPATHICS” model, a new framework in PP that advocates for a holistic view of both positive and negative emotions in L2 learning.

### 5.2 Contextual and methodological characteristics

In terms of the contextual characteristics of the reviewed studies, this study examined previous research on FLE from the perspectives of research countries, target languages, learning settings, participant characteristics, and sample sizes. Several patterns regarding the research contexts are worth noting.

First, one notable pattern regarding the distribution of countries is that most studies were conducted in China and Iran, focusing on FL learners who were native speakers of Chinese and Persian. This results in limited scholarly attention to the enjoyment of learners with other first languages. In light of cultural nonequivalence and conceptual nonequivalence regarding emotions (Li et al., 2018; Dewaele and Pavelescu, 2021), different languages may have distinct conceptual understandings of emotions. For example, Japanese people have a different understanding and conceptual structure of anxiety than Americans (Matsumoto, 1988). Therefore, the issue of cultural and conceptual nonequivalence of emotions in different countries with distinct languages points to a need to explore FLE among learners with diverse linguistic backgrounds in future research. Another striking result is that all the reviewed studies focused on EFL learners, which does not provide a comprehensive understanding of the FLE experience of learners of other target languages. Given that learners' target languages may affect their emotional experiences in language learning (De Smet et al., 2018), it is suggested that future studies on FLE should explore non-English learning settings to gain a more complete insight into FL learners' experiences of enjoyment.

Moreover, regarding the learning settings, most of the reviewed studies were still conducted in generic instructional settings, which may shed light on traditional in-person language learning and how foreign language (FL) learners experience foreign language education (FLE). However, there is very little research on FLE in other diverse instructional settings, such as online and blended learning contexts. FL learners may undergo different emotional developments in distinct settings. For example, the online classroom environment may affect learners' emotions by limiting physical and emotional interaction between teachers and students, as well as among students themselves (Marchand and Gutierrez, 2012). Therefore, more research should be undertaken to explore how different learning settings influence FLE. In terms of participant characteristics, university students constituted the dominant group of participants in the reviewed studies, resulting in a limited understanding of the FLE of secondary and primary school learners. As suggested by Peng et al. (2020), different levels of language learners may have varying emotional experiences in language learning. For example, Dewaele and Dewaele (2017) investigated enjoyment development in different age groups of secondary school students. The results revealed that FLE tended to increase in the older age group, corresponding with the findings of Dewaele and MacIntyre (2014), that older learners (ranging from pre-teens to participants in their sixties) reported a higher level of enjoyment. Therefore, the FLE across various age groups and language levels of learners warrants more scholarly attention in the future.

Regarding the methodological characteristics, one noteworthy result is that the majority of studies tended to adopt a quantitative research approach over qualitative and mixed methods. Although the predominant quantitative research methods have the advantage of allowing large-scale research on FLE within a short period, this methodology primarily examines learners' FLE from a static, linear, and monolithic perspective, which cannot capture the dynamic, complex, and situational characteristics of FLE at a micro level nor provide an in-depth description of the variations in learners' FLE (Li et al., 2024). Another remarkable pattern is that no study utilized qualitative research methods. In fact, qualitative methods and analysis tools, like thematic analysis, are particularly important in emotional research due to the highly subjective and fleeting nature of emotions, meaning they cannot be completely captured through quantitative approaches and statistical analysis. Therefore, more studies on FLE in the future are encouraged to utilize mixed methods, which could allow researchers to have binocular vision by combining diverse complementary data and perceiving the emotional episode in a holistic way (Dewaele, 2019). This is also in line with the call for a diversity of research methods in emotional research (MacIntyre, 2016).

Regarding the research designs, most studies employed cross-sectional designs, leaving longitudinal designs and other research methods under-researched. Although cross-sectional research designs facilitate the calculation of intergroup differences and can establish a model of the relationship between the FLE and other variables (Dewaele and Li, 2020), this approach does not enable researchers to capture the underlying causality and mechanisms of FLE. Additionally, while the cross-sectional approach is sufficient for exploring the synchronic emergence of FLE, it may be limited by the conceptualization of enjoyment as a stable trait that can be measured at a single time point (Jin and Zhang, 2018). Nonetheless, emotions display momentary variations due to the interplay and interaction among various variables in language learning (Elahi Shirvan et al., 2020). Therefore, more diverse research designs, such as the idiodynamic method and retrodictive qualitative modeling (RQM), should be employed in future studies to examine the development of enjoyment by collecting data across different time scales and considering the temporal properties of FLE, thus providing a more holistic understanding of the dynamics of enjoyment.

Regarding the data collection instruments, it is worth noting that self-report measures, such as questionnaires and open-ended surveys, were the most commonly used tools in FLE research. By self-rating on FLE measurement scales, the questionnaire instrument offers the advantages of simplicity, convenience, and low cost, especially in large-scale quantitative studies. Additionally, open-ended surveys and interviews, which were relatively underutilized qualitative methods in the reviewed studies, can help enrich the interpretation of qualitative findings. Despite their merits, self-report measures of FLE, which heavily rely on learners' subjective perceptions of FLE experiences, may face limitations in reliability and validity due to cognitive bias and social desirability during retrospective data collection (Dewaele and Li, 2020), resulting in a lack of objective data in this research. Therefore, future studies should consider adopting other types of instruments to collect data from diverse perspectives, such as observation, which can enable researchers to observe learners' verbal and non-verbal expressions of enjoyment, thus gaining a more in-depth understanding of enjoyment in specific learning situations.

### 5.3 Research gaps

This review makes great contributions to the field by identifying several gaps in current research on FLE in language learning from the PP. To begin with, given that the current research focuses on FLE and is mostly concerned with the relationship between FLE and other variables, as well as the predictors of FLE, there is a need to expand the research theme on FLE in the future, such as exploring emotional regulation strategies in language learning (Li and Xu, 2019; Mercer et al., 2018) and FLE in diverse language learning settings (Zheng and Zhou, 2022). Moreover, as the PP framework embraces theoretical plurality (MacIntyre et al., 2019), it is suggested that L2 researchers in enjoyment consider combining other theories, such as control-value theory (Pekrun, 2006), broaden-and-build theory (Fredrickson, 2001), and complex dynamic system theory (Cameron and Larsen-Freeman, 2007), to explore FLE from a holistic theoretical perspective (e.g., Ghafouri and Tahriri, 2023; Kruk et al., 2023). In addition, given the dynamic and complex nature of emotions, future inquiry warrants diversifying methodologies by integrating both quantitative and qualitative methods or adopting innovative research methods that possess both qualitative and quantitative characteristics, such as Q methodology (e.g., Kruk et al., 2022) and idiodynamic methods (e.g., Elahi Shirvan and Talebzadeh, 2018). Additionally, given the lack of qualitative methods, future studies should consider using qualitative methods such as written diaries, focused writing, and non-self-report measures like observation. Finally, more longitudinal designs for investigating FLE are needed in future research.

## 6 Limitations

This scoping review is not without limitations. One drawback is the limited number of studies reviewed (*n* = 36), confined solely to articles published in the WOS and SCOPUS databases. In addition, the exclusion of non-English language studies may have overlooked useful insights on this topic. Moreover, this review did not include review articles, conference proceedings, book chapters, and meta-analyses. Hence, future research should aim to address these issues to provide a more thorough investigation of this topic.

## 7 Conclusion

Enhancing language learners' emotional wellbeing and promoting their language learning have become significant concerns for L2 researchers and language teachers. Starting from this premise, this review aims to synthesize the current research foci on FLE, uncover the contextual and methodological features of existing research, and identify research gaps. The findings reveal that the current research themes of FLE primarily focus on exploring the relationship between FLE and various other variables, as well as the antecedents of FLE. Additionally, inquiries into FLE predominantly concern university students from China learning English in a generic classroom setting. These studies predominantly adopt cross-sectional quantitative methodologies. Finally, this review identifies research gaps that need to be addressed in the future. Taken together, this review provides valuable insights and implications for L2 researchers and language teachers to further explore this area of research.
